# Novel Expression of Apical Bile Acid Transport (ASBT) More Proximally Than Distal Ileum Contributing to Enhanced Intestinal Bile Acid Absorption in Obesity

**DOI:** 10.3390/ijms252111452

**Published:** 2024-10-25

**Authors:** Shanmuga Sundaram, Arunkumar Jagadeesan, Raja Singh Paulraj, Uma Sundaram, Subha Arthur

**Affiliations:** Department of Clinical and Translational Sciences, Joan C. Edwards School of Medicine, Marshall University, 1600 Medical Center Drive, Huntington, WV 25701, USA; sundaram1@marshall.edu (S.S.); jagadeesan@marshall.edu (A.J.); paulraj@marshall.edu (R.S.P.); sundaramu@marshall.edu (U.S.)

**Keywords:** obesity, obesity-related risk factors, metabolic disorders, bile acids, intestinal physiology, apical sodium bile acid transporter, farnesoid X receptor

## Abstract

Dietary lipid absorption is facilitated by bile acids. In the Zucker rat (ZR) model of obesity, bile acid absorption, mediated by the apical sodium bile acid transporter (ASBT), was increased in villus cells from the distal ileum. However, whether ASBT may be de novo expressed more proximally in the small intestine during obesity to facilitate additional bile acid absorption is not known. For this, starting from the end of the ileum to the mid jejunum, caudal-orally, five intestinal segments of equal length (S1–S5) were separated from lean and obese ZRs (LZR and OZR). Intestinal mucosa obtained from these segments were used for total RNA extraction, RT-qPCR and ^3^H-TCA uptake. The results showed that bile acid absorption along with the mRNA expression of ASBT and FXR progressively decreased caudal-orally in both LZRs and OZRs but was significantly higher in all small intestinal segments in OZRs. The expression of GATA4 was absent in the distal ileum (S1) in both LZRs and OZRs, but steadily increased along the proximal length in both. However, this steady increase was significantly reduced in the comparative obese proximal intestinal segments S2, S3, S4 and S5. The expressions of bile acid-activated G-protein-coupled bile acid receptor TGR5 and S1PR2 were unaltered in segments S1–S4 but were significantly increased in OZR S5. The paradigm changing observation of this study is that ASBT is expressed more proximally in the small intestine in obesity. This likely increases overall bile acid absorption and thereby lipid absorption in the proximal small intestine in obesity.

## 1. Introduction

Obesity is a growing epidemic with more than half of the global population (51%) predicted to be obese by 2035, with a global economic impact of $4 trillion (Obesity Atlas). In the United States, 42.4% of adults were reported to be obese in 2020 and accounts for 5.5–7.0% of national health expenditures [1]. Obesity is known to occur as a result of complex interactions between genetic, environmental, physiological, social and behavioral factors, and a wide variety of other biological factors that result in energy imbalance and promote excessive fat deposition [2,3,4,5,6,7,8]. Though the relative contribution of each of these factors has been studied extensively, the World Health Organization Consultation on obesity concluded that behavioral and environmental factors such as excess energy intake, especially dietary fat, are primarily responsible for the dramatic increase in obesity over the past two decades.

Dietary lipid digestion and absorption in the normal mammalian intestine is facilitated by bile acids that are synthesized as end products of cholesterol catabolism in the liver. A constant pool of bile acids is maintained in the enterohepatic circulation to aid the digestion and absorption of dietary lipids and lipid soluble vitamins. Though bile acids were traditionally known only for this function of facilitating dietary lipid absorption, studies in the last decade have recognized bile acids as vital signaling molecules implicating them in the regulation of glucose and lipid metabolism. Moreover, several studies have also demonstrated the molecular mechanisms underlying the complex interactions between intestine and liver bile acid signaling, and their impact on whole-body lipid, glucose and energy metabolism that leads to metabolic disorders [9,10,11,12,13]. In diet-induced obesity, increased dietary fat absorption has also been shown to lead to the disruption of bile acid and lipid homeostasis [14,15,16].

The principal intestinal participant in the enterohepatic circulation of bile acids is the apical sodium-dependent bile acid co-transporter (ASBT; SLC10A2) present in the brush border membrane (BBM) of distal ileal villus cells. ASBT plays a significant role in maintaining efficient enterohepatic circulation of bile acids and thereby enabling normal bile acid and lipid homeostasis. ASBT expression has been shown to play a significant role in several physiological and pathophysiological processes and are known to be regulated by numerous transcription factors and nuclear receptors as well as intestinal microbiota [17,18,19,20,21,22,23,24,25,26,27]. ASBT has been associated with the etiology and as the therapeutic target of inflammatory bowel disease, hepatobiliary diseases and several metabolic disorders such as Type 2 diabetes and hyperlipidemia [28,29,30,31,32,33,34,35,36].

Our previous studies conducted in a monogenic rat model of obesity (Zucker rat), a polygenic mouse model of obesity (TALLYHO mouse), and most importantly in obese human samples, showed that ASBT activity and expression were increased in the BBM of distal ileal villus cells. In addition, the cellular expression of bile acid-associated proteins, the ileal bile acid binding protein (IBABP) and organic solute transporter (OST), which are responsible for handling the transcellular transport of bile acids in villus cells, were also increased in Zucker rat model of obesity. This suggested increased transcellular transport of bile acids and therefore increased the efflux of bile acids from the villus cells to the portal vein. Furthermore, the expression of the bile acid-activated nuclear receptor, Farnesoid X receptor (FXR), was found to be enhanced in ileal villus cells and may likely be responsible for the increased ASBT expression at the cellular level in obesity [37]. In this same model of obesity, intestinal nutrient (glucose) and electrolyte (Cl) absorption were also found to be upregulated [38]. Since bile acids act as signaling molecules and are known to be vital regulators of epithelial function in health and disease [39], the upregulation of bile acid absorption by the epithelial cells in obesity could be vital in activating intracellular signaling mechanisms that might regulate other nutrient and electrolyte absorptive mechanisms that are altered in the intestinal epithelial cells in obesity.

Given this background, the novel hypothesis of this study is that ASBT-mediated bile acid absorption may be increased along the proximal length of the small intestine in addition to its known significant increase in the distal ileum to facilitate a net increase in intestinal bile acid absorption in obesity. Therefore, the aim of the study was to determine the alterations in bile acid absorption, ASBT expression and the expression of its known regulatory proteins along the length of the small intestine in obese Zucker rats. It is important to determine the absorptive capacity of the small intestine in obesity since the increase in the net absorption of bile acids might facilitate the rapid onset and progression of obesity and its associated disorders such as dyslipidemia.

## 2. Results

### 2.1. Altered Bile Acid Absorption along the Length of the Small Intestine in the Zucker Rat Model of Obesity

**Figure 1:**
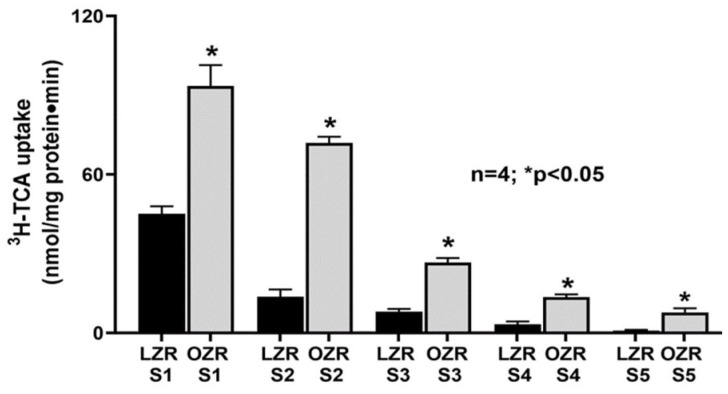
^3^H-TCA uptake was significantly higher in all intestinal segments in OZRs compared to their relative segments in LZRs.

**Figure 2:**
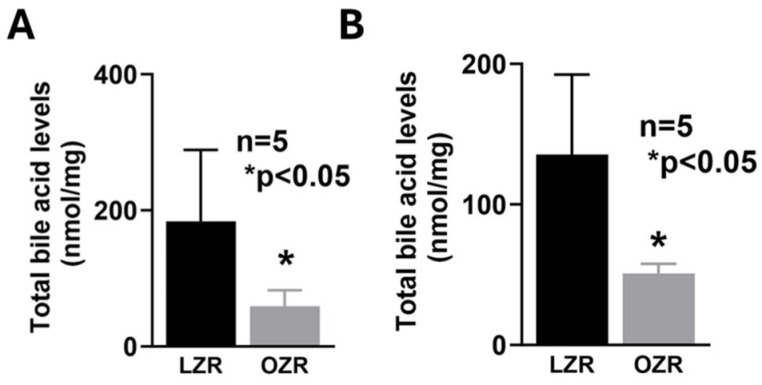
Total bile acid levels were significantly decreased in the intestinal content obtained from the (**A**) distal ileum and the (**B**) cecum in OZRs compared to LZRs.

BBM vesicle (BBMV) functional studies showed that the ^3^H-TCA uptake was highest in the S1 segments in both lean ZRs (LZRs) and obese ZRs (OZRs). However, ^3^H-TCA uptake was higher in S1 of OZR compared to S1 of LZR, substantiating the ASBT mRNA expression data and the cellular data published in our previous study [37]. Moreover, ^3^H-TCA uptake was significantly higher from S2 to S5 in OZRs compared to their relative segments in LZRs, though ^3^H-TCA uptake gradually decreased in both LZR and OZR segments S2 through S5 (Figure 1). Moreover, the total bile acid levels were significantly decreased in the intestinal content obtained from the distal ileum and the cecum in OZRs compared to LZRs (Figure 2), indicating increased bile acid absorption along the proximal length of the small intestine in obesity resulting in decreased bile acid levels entering the distal ileum and the cecum.

### 2.2. Altered Expression of ASBT and Its Regulatory Protein mRNA Along the Length of the Small Intestine in the Zucker Rat Model of Obesity

**Figure 3:**
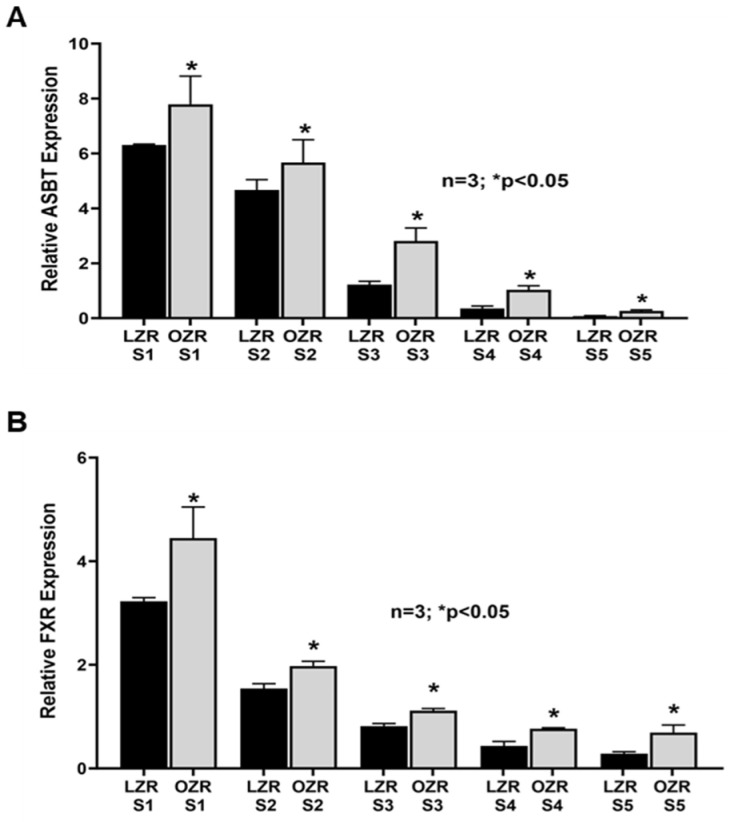
(**A**) ASBT expression was significantly increased in all segments along the caudal-oral length of the small intestine in obesity. (**B**) FXR mRNA expression was highest in the distal ileal segments (S1) and decreased along the caudal-oral length of small intestine in both LZRs and OZRs. However, compared to LZR small intestinal segments, FXR expression was increased in all segments in OZRs.

**Figure 4:**
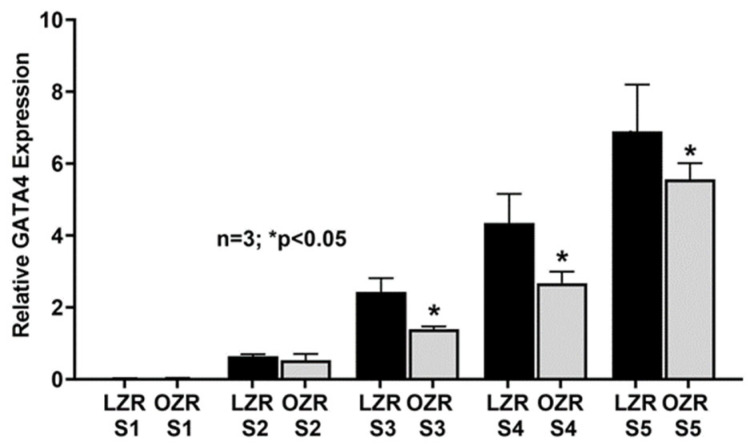
GATA4 mRNA expression was absent in the distal ileum and increased along the caudal-oral length as expected in LZRs and OZRs. However, its expression was decreased in proximal segments in OZRs compared to LZRs.

ASBT’s expression was highest in the distal ileum (S1) of both LZRs and OZRs. However, the expression of ASBT was significantly higher in the distal ileum of OZRs compared to LZRs, as expected. In both LZRs and OZRs, ASBT’s expression progressively decreased caudal-orally in both LZRs and OZRs but was significantly higher in all the small intestinal segments obtained from the OZR intestine compared to their comparative segments in LZRs (Figure 3A). ASBT expression in the distal ileum has been shown to be regulated by several transcription factors. Therefore, we performed RT-qPCR of two known transcriptional regulators of ASBT such as FXR, known to regulate ASBT expression at the distal ileum, and GATA4, which is known to induce the expression of ASBT at the proximal intestine. We found that FXR expression was significantly increased in the distal ileal segment S1 in obesity, thus confirming our cellular data from the distal ileum of OZRs (Figure 3B). Moreover, FXR expression increased significantly in all the other proximal segments compared to its corresponding LZR proximal small intestinal segments. As shown in Figure 4, GATA4 expression was absent in the distal ileum (S1) in both LZRs and OZRs, where ASBT expression is at its highest. Moreover, its expression steadily increased along the proximal length of the LZR small intestine. But this steady increase in GATA4 expression was significantly reduced in the comparative proximal intestinal segments S2, S3, S4 and S5 of OZRs. 

**Figure 5:**
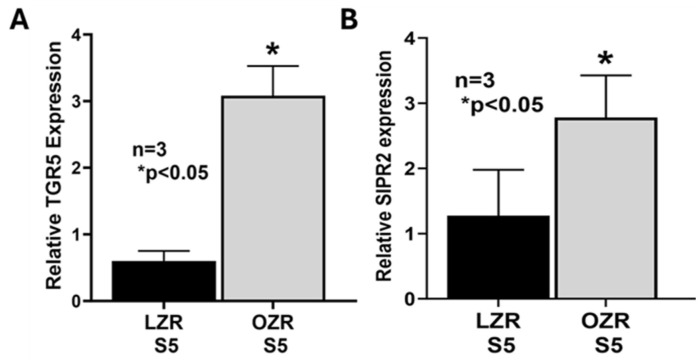
mRNA expression of bile acid receptors (**A**) TGR5 and (**B**) S1PR2 were increased in mid-jejunal segments (S5) of OZRs compared to LZRs.

Interestingly, in addition to the increased expression of ASBT, GATA4 and FXR, expression of bile acid-activated G-protein-coupled bile acid receptors TGR5 (Gpbar1; Figure 5A) and S1PR2 (Sphingosine-1-Phosphate Receptor 2; Figure 5B) were increased only in the villus cells of proximal small intestine (S5) in OZRs and remained unchanged between LZRs and OZRs in segments S1 to S4.

## 3. Discussion

Bile acids and their intestinal absorption mediated by ASBT have been implicated in the pathophysiology of obesity and several metabolic disorders including dyslipidemia [40,41]. Though the importance of ASBT in obesity is well accepted, the findings of the present study where ASBT has been shown to be expressed also in the proximal small intestine in obesity is very significant since ASBT was known hitherto to be expressed only in the distal ileum of the small intestine [42]. This novel finding was further substantiated by increased bile acid absorption along the caudal oral length of the small intestine, indicating that this net increase in ASBT-mediated bile acid absorption may play a vital role in the pathogenesis of obesity. This was further substantiated by significant reduction in bile acid levels in OZRs compared to LZRs, both in the distal ileum as well as in the cecum, indicating that bile acids are absorbed actively throughout the small intestine, resulting in reduced levels entering the distal intestine and cecum. Given these novel observations of increased bile acid absorption and increased ASBT expression along the small intestine in obesity, it is perhaps not surprising that non-specific treatments such as bile acid sequestrants have not been a universally useful treatment option for obesity-associated dyslipidemia. This could be due to the inability of the recommended dosage of the sequestrants to completely clear the elevated levels of bile acids secreted in the intestine in obesity and/or its ability to cause undesirable side effects such as gastrointestinal symptoms, vitamin deficiency, interference with other drug absorption, transient hypertriglyceridemia, etc. [43], when used long term.

Various studies have previously shown that expression of ileal ASBT and other intestinal bile acid transporters are significantly elevated in rats with streptozocin-induced diabetes mellitus [44,45], specifically by increased ASBT promoter activity [45]. Moreover, increased ASBT expression was accompanied by increased bile acid pools in these rats. These studies demonstrate that ASBT expression is highly regulated by transcriptional mechanisms. Thus, in the context of this study, it is clear that ASBT expression is transcriptionally regulated during obesity. In this study, the increased ASBT expression might be facilitated by altered GATA4 and/or FXR expressions along the length of the small intestine in obesity. Though GATA4 and FXR show differential expressions along the length of the OZR small intestine, the pattern of differential expression is diametrical with each other. Interestingly, it has been previously shown that the expression of FXR and its downstream-regulated genes such as ASBT are upregulated in the GATA4 null mice jejunum [46], which suggests that GATA4 might regulate the altered expression of both ASBT and FXR along the proximal length of the small intestine in obesity. Alternatively, ASBT upregulation might be regulated either by the upregulation of FXR expression or downregulation of GATA4 along the length of the small intestine in obesity.

In a mouse model, attenuation of gut microbiota has been shown to alter the expression of ASBT by GATA4, increasing the intestinal absorption of bile acids [26]. More related to the data obtained in the present study, few previous studies have shown that intestinal resection increases ASBT expression in the remainder of the intestine as an adaptive mechanism [24,47,48]. Further, these studies noted that transcription factor GATA4 may play a role in the altered expression of ASBT in the unresected intestine. For example, it was shown that while the downregulation of GATA4 induced the expression of ASBT, the induction or expression of GATA4 repressed ASBT [46,49]. These studies suggest that the GATA4 induction in the proximal length of the OZR small intestine is likely an adaptive physiological mechanism to absorb the increased bile acid levels in the small intestine.

ASBT expression and activity are also known to be modulated by the nuclear receptor FXR in the intestinal epithelial cells [50,51]. FXR is a well-known bile acid-activated nuclear receptor protein which is also known to transcriptionally regulate genes involved in maintaining bile acid homeostasis [52,53,54]. In our previous study, we demonstrated that FXR levels were significantly increased in the distal ileal villus cells in OZRs. Moreover, FXR activation in vitro using an FXR agonist resulted in the upregulation of ASBT and bile acid transporting proteins, the ileal bile acid binding protein and organic solute transporter, leading to increased transepithelial transport of bile acids in the intestine [37]. Therefore, in this study, the increase in the expression of ASBT along the proximal length of the OZR small intestine is either regulated by simultaneous increases in FXR expression or by a diametrically opposite mechanism mediated by GATA where its downregulation along the proximal length of the OZR small intestine might induce the upregulation of ASBT expression.

The mid-jejunal segment (S5) showed significant levels of ASBT expression indicating that jejunum might play a role in the net increases in bile acid absorption in the intestine in obesity. In addition, there was a significant increase in the expression of the bile acid receptors TGR5 and S1PR2 only in the mid-jejunal segment S5, indicating that the enterocytes at this region of OZR intestine might be differently regulated compared to the molecular regulating mechanisms in the other segments. TGR5 and S1PR2 are G-protein-activated receptors (GPCRs) and are known to activate several downstream kinase enzymes and pathways [55,56]. One common signaling pathway that is known to be activated by their activation is the cAMP-activated PKA pathway which has been implicated in obesity and associated disorders. Therefore, future studies will be conducted to determine the GPCR-mediated downstream signaling pathways that regulate increased bile acid absorption in the mid-jejunal segment of the OZR small intestine. The knowledge gained from these studies will not only provide a comprehensive knowledge of the regulatory pathways of altered intestinal bile acid absorption in obesity but will also provide novel intestine specific therapeutic targets to treat obesity and its associated dyslipidemia.

One limitation of the present study is that the studies were conducted in a species that lacks gall bladder. However, gall bladder is not an essential organ. Rats that lack a gall bladder and patients who have undergone cholecystectomy are still able to absorb lipids from the diet due to direct secretion of bile into the duodenum [57]. Moreover, rats are an ideal animal model to study intestinal physiology and pathophysiology with regards to understanding ASBT regulation since they express ASBT only in the distal ileum in normal conditions as in the human intestine. Most importantly, we confirmed in our previous study that ASBT expression was upregulated in the distal ileum of OZRs and this was comparable to the ASBT data obtained from ileal biopsies of obese subjects, thus showing that our data from the rat model of obesity is translationally relevant and significant, making them an ideal model to study ASBT regulation in obesity [37].

## 4. Materials and Methods

### 4.1. In Vivo Model of Obesity

Zucker rats served as the in vivo model of obesity (males at 18 weeks of age). Obese Zucker rats (OZRs; a monogenic model of obesity) show leptin resistance (*Lepr^fa^*), which is strongly associated with extreme obesity and is a heritable trait. OZRs are characterized by hypertriglyceridemia, hypercholesterolemia and metabolic syndrome associated with morbid obesity. Lean Zucker rats (LZRs) served as controls. LZRs and OZRs obtained from Charles River Laboratories International, Inc. (Wilmington, MA, USA) were included in the study after a week of acclimatization in the animal facility. They were maintained in the rat housing facility in a light and dark cycle (12 h/12 h), with regulated temperature and humidity conditions and unrestricted access to food and water. All the experimental procedures with the Zucker rats were then carried out strictly in accordance with the procedural and ethical regulations of Marshall University’s Institutional Animal Care and Use Committee (IACUC approval # 756).

### 4.2. Intestinal Segments and Mucosa Preparation

**Figure 6:**
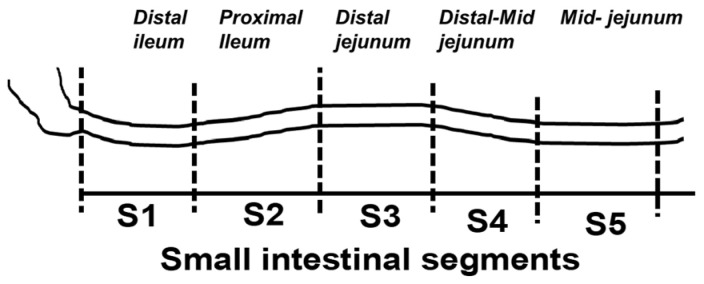
Illustration of segmental separation of small intestine to study alterations in the distribution of ASBT expression and its regulatory proteins.

Starting from the end of the ileum moving caudal-orally to the mid jejunum, five intestinal segments of equal length (6 inches) were demarcated and separated as S1, S2, S3, S4 and S5 and these five segments constituted 80% of the total length of the intestine (Figure 6). Intestinal mucosa was separated from these segments by scrapping. Scrapped mucosa from each of these segments were either flash frozen for uptake experiments or were used for total RNA extraction.

### 4.3. BBM Vesicle Preparation and ^3^H-Taurocholate Uptake Studies

BBM vesicles (BBMVs) were prepared with scrapped mucosa using Mg^++^ precipitation and the differential centrifugation method [58,59]. BBMVs were then suspended in an incubation buffer containing 100 mM choline chloride, 0.1 mM MgSO_4_, 50 mM HEPES-Tris (pH 7.5), 50 mM mannitol and 50 mM KCl. After an hour of incubation, the BBMV suspension was used for ^3^H-taurocholate (TCA) uptake experiments as described previously [37].

### 4.4. Bile Acid Measurement

The intestinal content from the distal ileum and the cecum were collected from LZRs and OZRs and were sent to the Metabolomics Core Facility, University of Michigan, for bile acid level measurements. Bile acids were extracted by two-step solvent extraction, separated using liquid chromatography–mass spectrometry and measured by the ESI- QQQ MRM method.

### 4.5. Real Time-Quantitative PCR (RT-qPCR) Analysis

Total RNA was extracted from the mucosa using the RNeasy mini kit (Qiagen, Cat#74104, Hilden, Germany) and the genomic DNA was digested using RNase-Free DNase (Qiagen, Cat# 79256, Hilden, Germany). Single-step high-sensitivity quantitative PCR was performed using Brilliant III Ultra-Fast SYBR Green qRT-PCR Master Mix (Agilent, Cat# 600886, Santa Clara, CA, USA). Rat-specific exon–exon spanning oligonucleotide primers were generated by NCBI BLAST (Table 1) and chemically synthesized by the Invitrogen custom primer service (Thermo Fisher Scientific, Waltham, MA, USA) and optimized for their target specificity by melt curve analysis and product length determination by agarose gel electrophoresis. The rat-specific beta-actin was used as an endogenous control to normalize the expression of targets.

### 4.6. Protein Quantification

Protein quantitation for BBM uptake experiments and Western blot studies were performed using Lowry’s method with the DCTM protein assay kit (Bio-Rad, Berkeley, CA, USA), according to manufacturer’s instructions.

### 4.7. Statistical Analysis

The data included in this study were calculated with the GraphPad Prism 9.1.0 (San Diego, CA, USA) software and are expressed as means ± SEM. The “n” number in each data set indicates that both the functional and molecular experiments were performed with cells isolated from different animals, each n indicating one animal. Also, each “n” in the uptake experiment is an average of uptakes performed as a triplicate. Total bile acid data were analyzed using unpaired Student’s *t*-test. Uptake data generated in this study were analyzed using the Wilcoxon–Mann–Whitney test. RT-qPCR data were analyzed using two-way analysis of variance (ANOVA, Tukey’s multiple comparisons test) using GraphPad Prism 9.1.0.

## 5. Conclusions

This study demonstrates ASBT-mediated net increases in intestinal bile acid absorption along the proximal length of the small intestine in obesity, which is likely responsible for the rapid onset and progression of obesity and its associated dyslipidemia. Furthermore, this study also provides a comprehensive insight into bile acid-activated signaling mechanisms that may play a significant role in increased ASBT-mediated bile acid absorption in the proximal small intestine. Finally, the novel observation of increased expression of ASBT along the proximal small intestine beyond the distal ileum as seen in this study is very significant and a heretofore observation unknown in intestinal physiology.

## Figures and Tables

**Table 1:** Rat-specific exon–exon spanning oligonucleotide primers used for the specific target gene.

Target	Forward Primer	Reverse Primer
*ASBT*	TCGCAGGTGCAATTCTCATTGT	CCAAGGCAACTGTTCGGCAC
*FXR*	CAAGCCACGGACGAGTTTGC	CAGTCTTCCGGTTGTTGCGG
*GATA4*	CCTGCGAGACACCCCAATCT	GTCCTGTCCCATCTCGCCTC
*TGR5*	GCCCAAAGGTGGCTACAAGT	GCATTGGCTACTGGAGTGGT
*SIPR2*	TGCTGCCCCTCTATGCTAAG	GGCCACGATAGCCAGTAAGA
*Beta-Actin*	ACGGTCAGGTCATCACTATCGG	TGTAGTTTCATGGATGCCACAGGAT

## Data Availability

For verifiable query, data will be provided to investigators actively engaged in intestinal transport physiology research.
